# A LASSO regression-driven nomogram to predict functional outcomes after surgery for chronic lateral ankle instability

**DOI:** 10.3389/fsurg.2026.1758259

**Published:** 2026-08-05

**Authors:** Feng Lin, Zhiyao Lv, Zhidong Zhao, Yulei Gao

**Affiliations:** 1 Department of Joint Surgery, Central Hospital Affiliated to Shandong First Medical University, Jinan, China; 2 Department of Orthopedics, Chinese PLA Hospital, Beijing, China

**Keywords:** arthroscopic broström repair with suture-tape augmentation, chronic lateral ankle instability, nomogram model, risk factors, suture-tape augmentation

## Abstract

**Objective:**

This study aimed to identify predictors of postoperative recovery in chronic lateral ankle instability (CLAI) and construct a risk-estimation nomogram for individualized risk assessment.

**Methods:**

Between January 2021 and December 2023, 132 patients with CLAI who underwent arthroscopic Broström repair with suture-tape augmentation were retrospectively included and classified into good-recovery [American Orthopaedic Foot and Ankle Society (AOFAS) ankle-hindfoot score ≥75, *n* = 73] and poor-recovery groups (AOFAS <75, *n* = 59). Candidate predictors were identified using univariate analysis, LASSO regression, and multivariable logistic regression. A nomogram was developed and internally validated using 1,000 bootstrap resamples, and model performance was assessed by ROC curve analysis, calibration analysis, and decision curve analysis (DCA).

**Results:**

Six variables were identified as independently associated with poor postoperative recovery: sprain count (OR = 1.439, *P* = 0.043), osteophytes (OR = 5.951, *P* = 0.004), syndesmosis injury (OR = 5.272, *P* = 0.007), Outerbridge grade II cartilage damage (OR = 23.928, *P* = 0.017), thin or absent ATFL remnant <1.0 mm (OR = 5.256, *P* = 0.029), and CFL/ATFL angle <70° (OR = 11.598, *P* = 0.003). The nomogram showed acceptable discrimination, with an apparent AUC of 0.907 and an optimism-corrected AUC of 0.876 after 1,000 bootstrap resamples. Calibration demonstrated acceptable agreement between predicted and observed outcomes, and DCA suggested potential clinical utility.

**Conclusion:**

This study identified six variables associated with AOFAS-defined poor postoperative functional recovery in patients with CLAI and developed an internally validated nomogram for individualized risk estimation.

## Introduction

Chronic lateral ankle instability (CLAI) is a common condition that may substantially restrict daily activities (1). The clinical manifestations of CLAI extend beyond mechanical laxity and include persistent proprioceptive deficits, impaired neuromuscular control, and reduced dynamic postural stability (2). These abnormalities predispose patients to recurrent episodes of ankle instability, persistent pain, and functional impairment. More importantly, long-term biomechanical abnormalities may accelerate degenerative changes within intra-articular structures, thereby increasing the risk of osteochondral lesions of the talus (OLTs) and post-traumatic osteoarthritis (PTOA), which can ultimately lead to substantial long-term disability (3–5). For patients with CLAI who remain symptomatic despite 3–6 months of systematic non-surgical treatment, surgical intervention is widely accepted as an effective strategy for restoring ankle stability and function (6). At present, modified Broström repair augmented with an internal brace or suture-tape reinforcement is commonly used in the surgical management of CLAI. This technique offers several advantages, including reliable short-term clinical efficacy, limited iatrogenic injury, and accelerated postoperative rehabilitation (7). Nevertheless, despite generally favorable outcomes, a subset of patients still fail to achieve satisfactory functional recovery after surgery and may experience residual pain, recurrent instability, or persistent functional limitation (8, 9).

The marked inter-individual variability in postoperative outcomes highlights the need to identify key prognostic factors influencing surgical recovery in patients with CLAI. Such information may facilitate more accurate preoperative risk stratification and support the development of individualized treatment strategies. Previous studies have reported that poor postoperative outcomes in patients with CLAI are associated with higher body mass index (BMI), generalized ligamentous laxity, frequent ankle sprains, longer disease duration, and concomitant intra-articular or periarticular lesions, including OLTs, distal tibiofibular syndesmotic instability, and anterolateral ankle impingement with osteophyte formation (10–12). However, most existing studies have focused on individual risk factors, leading to relatively fragmented conclusions and limited insight into the combined effects and relative importance of multiple prognostic variables (13).

A clinically applicable multivariable predictive tool that integrates multidimensional preoperative information to identify patients at high risk of poor functional recovery after surgery for CLAI remains lacking. In this study, the least absolute shrinkage and selection operator (LASSO) regression was used for variable selection, and a clinically applicable nomogram was constructed to predict poor postoperative functional recovery. The model performance was evaluated in terms of discrimination, calibration, and clinical utility, and its robustness was assessed using internal validation. We hypothesized that the proposed nomogram could provide a useful preoperative tool for estimating the risk of poor functional recovery after CLAI surgery and supporting individualized clinical decision-making.

## Materials and methods

### Patients inclusion

This retrospective cohort study was conducted in accordance with the Declaration of Helsinki and was approved by the Medical Ethics Committee of the Chinese PLA General Hospital (approval No. 2025KY33-KS001). Written informed consent was obtained from all participants before data collection. To protect participant privacy, all identifiable personal information was de-identified during data collection and analysis. Patients diagnosed with CLAI who underwent arthroscopic Broström repair with suture-tape augmentation in the Department of Sports Medicine at our hospital between January 2021 and December 2023 were included.

The inclusion criteria were as follows: (1) a definitive diagnosis of CLAI, characterized by recurrent ankle sprains, subjective ankle instability or giving-way episodes, pain, swelling, or functional limitation after the initial sprain, in accordance with established diagnostic criteria for CLAI (14). This criterion ensured that all enrolled patients had clinically relevant chronic instability rather than isolated acute ligament injury; (2) preoperative magnetic resonance imaging (MRI) evidence of anterior talofibular ligament (ATFL) tear, with intact calcaneofibular ligament (CFL) and posterior talofibular ligament (PTFL). This criterion was applied to include patients with ATFL-dominant lateral ankle instability who were suitable for arthroscopic Broström repair with suture-tape augmentation, while minimizing heterogeneity caused by more extensive lateral ligament complex injuries requiring different surgical strategies; (3) treatment with arthroscopic Broström repair with suture-tape augmentation according to a standardized operative protocol, to reduce variability related to surgical technique; (4) a minimum follow-up duration of 12 months with available American Orthopaedic Foot and Ankle Society (AOFAS) Ankle-Hindfoot scores, as this period was considered necessary for evaluating postoperative functional recovery after ligament repair; and (5) complete clinical, imaging, intraoperative, and follow-up data required for reliable predictor assessment and model construction. The exclusion criteria were as follows: (1) previous surgery or revision surgery on the affected ankle, because prior surgery may alter local anatomy, ligament quality, scar formation, and rehabilitation response, thereby confounding the assessment of postoperative recovery; (2) preoperative MRI evidence of large talar cartilage defects measuring ≥15 mm. Large osteochondral lesions may independently cause pain, swelling, and functional limitation and may require additional cartilage repair procedures, making AOFAS-defined postoperative recovery less attributable to ligament repair alone; (3) obvious subtalar joint instability, because this condition can mimic or aggravate lateral ankle instability and may require different surgical management; (4) foot deformities or abnormal lower-limb alignment, including flatfoot deformity or abnormal mechanical axis, which may alter ankle biomechanics and affect postoperative functional recovery; (5) systemic inflammatory disease, neuromuscular disease, or other disorders that could affect ligament healing, muscle control, proprioception, or rehabilitation; (6) postoperative wound infection, poor wound healing, or the need for additional surgery during the treatment period, as these complications may directly affect postoperative function and introduce outcome bias; and (7) incomplete follow-up data due to refusal of follow-up or loss of contact, because complete follow-up data were necessary for reliable outcome classification and model development.

### Surgical procedure and standardization of intraoperative recording

**Figure 1:**
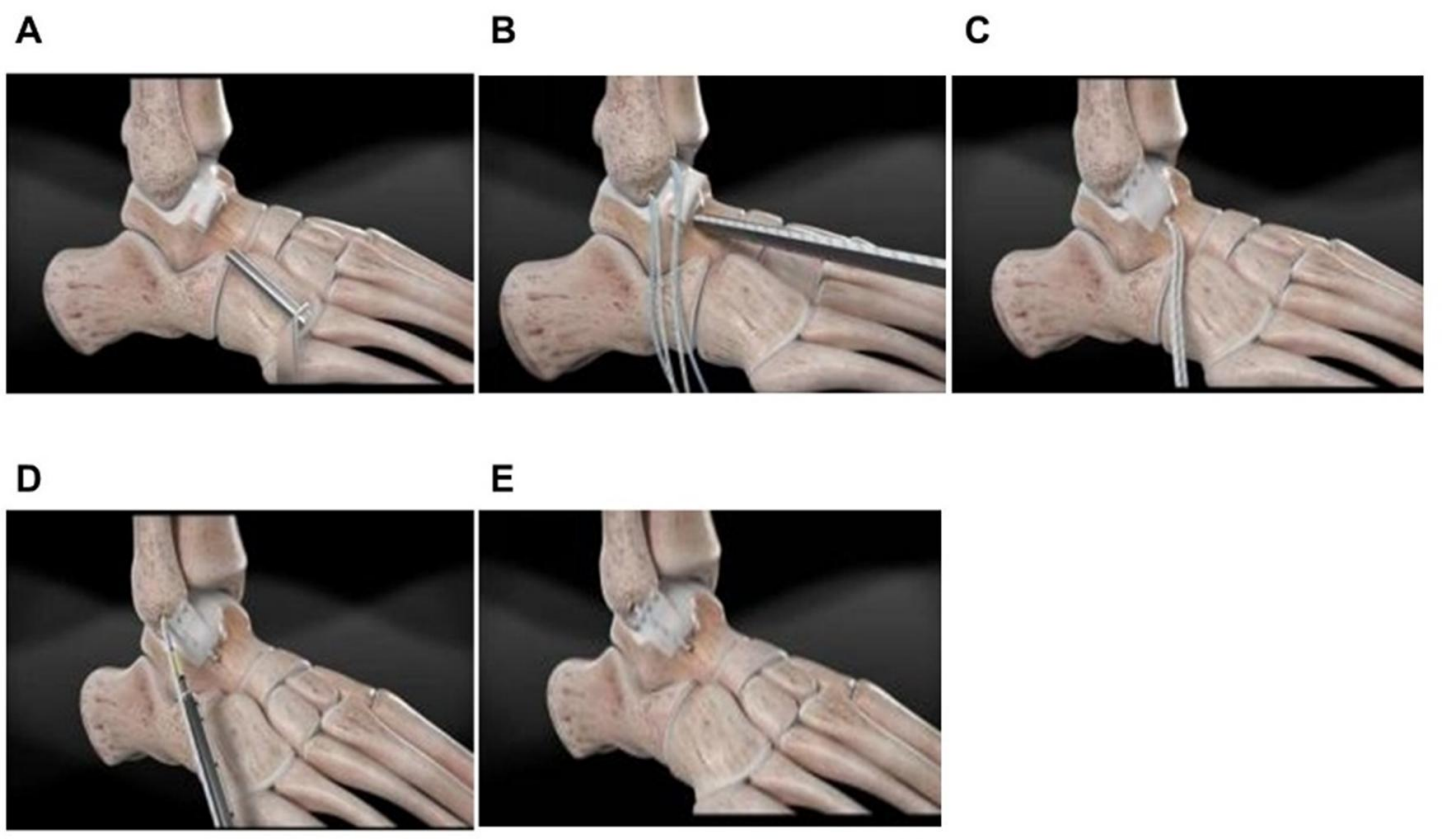
Surgical schematic of arthroscopic modified broström-gould repair with suture-tape augmentation. **(A)** A knotless anchor loaded with suture tape was placed at the talar attachment site. **(B)** The fibular attachment site was refreshed, and an anchor was inserted into the fibular footprint. **(C)** The anterior talofibular ligament (ATFL), joint capsule, and extensor retinaculum were repaired using an overlapping suture technique. **(D)** The suture tape was fixed on the fibular side using an external-row anchor under appropriate tension. **(E)** The remaining suture ends were trimmed after final fixation.

All arthroscopic modified Broström-Gould repairs with suture-tape augmentation were performed by the same senior orthopedic surgeon specializing in sports medicine and foot and ankle surgery. The surgeon had extensive experience in lateral ankle ligament repair and routinely performed this procedure before the study period. To minimize technical variability, all surgeries were conducted according to a standardized operative protocol. Briefly, a knotless anchor loaded with suture tape was first placed at the talar attachment site. The fibular footprint of the lateral ligament complex was then refreshed, and an anchor was inserted at the fibular attachment site. The ATFL, joint capsule, and extensor retinaculum were repaired using an overlapping suture technique. The suture tape was subsequently fixed to the fibular side using an external-row anchor, followed by controlled tensioning and trimming of the remaining suture ends (Figures 1A–E). Ankle stability was assessed intraoperatively after ligament repair and suture-tape augmentation. Intraoperative findings were documented immediately after surgery using a predefined operative record form. The recorded variables included talar cartilage status, anterior ankle osteophyte formation, syndesmotic injury, ATFL remnant quality, and other concomitant intra-articular abnormalities. Talar cartilage injury was evaluated according to the Outerbridge classification: grade 0, normal cartilage; grade I, cartilage softening or swelling; grade II, fibrillation or superficial fissures not extending to the subchondral bone; grade III, fissuring extending to the subchondral bone; and grade IV, exposed subchondral bone. Anterior ankle osteophyte formation was recorded as present when bony impingement or osteophyte formation was identified at the anterior ankle during surgical inspection. Syndesmotic injury was determined based on preoperative imaging findings combined with intraoperative assessment of syndesmotic stability. ATFL remnant quality was assessed according to intraoperative inspection and preoperative magnetic resonance imaging findings. To improve data reliability, intraoperative findings were independently extracted from the operative records by two researchers. Any disagreement regarding data extraction or grading was resolved through discussion with the operating surgeon or a senior foot and ankle specialist.

### Postoperative rehabilitation and follow-up

All patients received the same standardized postoperative ankle rehabilitation protocol. An ankle brace was used for 4 weeks after surgery, with continuous use at night and intermittent daytime use as needed; patients were instructed to avoid weight-bearing during this early protective phase. At 1–2 weeks postoperatively, passive dorsiflexion and active plantarflexion exercises were initiated. After 2 weeks, active and passive range-of-motion exercises in multiple directions, together with peroneal muscle strengthening, were gradually introduced. Progressive weight-bearing was started at 4 weeks, and by 8 weeks patients were generally allowed to perform basic daily activities under full weight-bearing conditions. Gradual return to sports was permitted after 3 months according to functional recovery and clinical evaluation.

### Data collection and outcome evaluation

Demographic and clinical data were collected, including sex, age, BMI, affected side, Beighton score for generalized ligamentous laxity, number of ankle sprains, disease duration, and other relevant clinical variables. Imaging parameters, including the ATFL-CFL angle (15) ATFL remnant thickness (16), subchondral bone marrow edema grade (17), and preoperative MRI-based joint effusion grade (18), were assessed using preoperative MRI. All patients were followed for at least 12 months. Postoperative functional recovery was assessed at the final follow-up using the AOFAS Ankle-Hindfoot score. The AOFAS score assesses three domains, including pain, ankle joint function, and alignment (19). Scores were categorized as excellent for 90–100 points, good for 75–89 points, fair for 50–74 points, and poor for <50 points (20–22). The categorical and ordinal grading criteria used in this study is summarized in Supplementary Table S1.

### Development and internal validation of the nomogram model

This study should be interpreted as a model-development study. Given the limited sample size and the relatively large number of candidate clinical, imaging, and intraoperative variables, a pragmatic stepwise variable-reduction strategy was used. Univariate logistic regression was first performed as an initial screening step to reduce the number of candidate variables associated with AOFAS-defined poor postoperative functional recovery. Variables with *P* < 0.05 were then entered into least absolute shrinkage and selection operator (LASSO) regression with 10-fold cross-validation to further optimize variable selection and reduce potential multicollinearity. LASSO regression was performed using the “glmnet” package in R software (version 4.2.1; R Foundation for Statistical Computing, Vienna, Austria). Variables with non-zero coefficients were subsequently included in a multivariable logistic regression model. Odds ratios (ORs), 95% confidence intervals (CIs), and *P*-values were reported to facilitate clinical interpretation and nomogram construction. We acknowledge that this variable-selection pipeline is not a purely standard prediction-modeling strategy. In particular, univariate pre-screening before LASSO may introduce selection bias, and refitting an unpenalized multivariable logistic regression model after penalized variable selection may produce inflated estimates, especially for sparse categorical predictors. Therefore, Firth penalized likelihood logistic regression was additionally performed as a sensitivity analysis to reduce small-sample bias and evaluate the robustness of the final model estimates, particularly for categories with sparse cell counts or possible quasi-complete separation. The Firth-corrected ORs, 95% CIs, and *P*-values were reported and compared with the conventional logistic regression results. A risk-estimation nomogram was constructed based on the final multivariable logistic regression model using the “rms” package in R software. Model discrimination was evaluated using receiver operating characteristic (ROC) curve analysis and the area under the ROC curve (AUC). Internal validation was performed using 1,000 bootstrap resamples to estimate optimism-corrected model performance. The mean optimism in AUC was calculated, and the optimism-corrected AUC was reported. Model calibration was assessed using calibration curves, calibration intercept, calibration slope, mean absolute error, and the Hosmer–Lemeshow goodness-of-fit test. Decision curve analysis (DCA) was applied to evaluate the potential clinical utility of the nomogram across different threshold probabilities.

Although the final nomogram incorporated six clinical predictor domains, the effective model complexity was evaluated according to the number of non-intercept regression coefficients estimated in the final logistic regression model. Accordingly, the final logistic regression model estimated nine non-intercept coefficients. Given that 59 patients experienced AOFAS-defined poor postoperative functional recovery, the events-per-variable calculated according to the effective degrees of freedom was 59/9 = 6.56. This value was below the conventional threshold of 10 events per variable and was considered when interpreting model stability and overfitting risk.

### Statistical analysis

All statistical analyses were performed using SPSS version 29.0 (IBM Corp., Armonk, NY, USA) and R software. Continuous variables with a normal distribution were expressed as the mean ± standard deviation (SD) and compared between groups using the independent-samples t-test. Non-normally distributed continuous variables were presented as the median and interquartile range [M (P25, P75)] and compared using the Mann–Whitney U test. A *p*-value <0.05 was considered statistically significant. Categorical variables were expressed as numbers and percentages. For between-group comparisons of categorical variables, Fisher's exact test was used for 2 × 2 contingency tables, and the Fisher-Freeman-Halton exact test was used for R × C contingency tables. All exact tests were performed using direct exact calculation without Monte Carlo approximation. A two-sided *P*-value <0.05 was considered statistically significant.

## Results

### Baseline characteristics of the study cohort

A total of 132 patients diagnosed with CLAI were included in this study (Supplementary Table S2). The cohort consisted of 113 males (86.0%) and 19 females (14.0%), indicating a predominantly male study population. Most patients were classified into age group 2 (92.0%), whereas only a small proportion belonged to the other age categories. Assessment of generalized ligamentous laxity showed a broad distribution of Beighton scores: 41.0% of patients had a score of 2, 20.0% had a score of 1, and 19.0% had a score of 0, while fewer patients had higher scores (≥3). Regarding sprain history, most patients had experienced 2–4 ankle sprains (67.0%), whereas 8.3% had experienced only one sprain and 9.1% reported ≥7 sprains. The mean injury duration was 17.2 ± 20.1 months, suggesting substantial chronicity within the cohort. Syndesmotic injury was observed in 43 patients (33.0%), whereas 89 patients (67.0%) showed no evidence of syndesmotic injury. Arthroscopic cartilage grading showed that 57.0% of patients had normal talar cartilage, 32.0% had cartilage softening corresponding to Outerbridge grade I, and 11.0% had fibrillation or superficial fissures not extending to the subchondral bone, corresponding to Outerbridge grade II. Regarding MRI-derived ATFL remnant thickness, 31.0% of patients were within the normal range (1.0–3.2 mm), 39.0% had thickened remnants (>3.2 mm), and 30.0% had thin or absent remnants (<1.0 mm). Bone marrow edema grading revealed that 40.0% of patients had no edema, 33.0% had small bone marrow edema, and 27.0% had large bone marrow edema. Similarly, joint effusion was absent in 36.0% of patients, whereas 43.0% had effusion occupying less than 50% of maximal capsular distension, and 21.0% had effusion occupying 50% or more of maximal capsular distension.

The CFL/ATFL angle, reflecting ligament alignment, was ≥91° in 22.0% of patients, 70°–90° in 25.0%, and <70° in 53.0%. Based on the AOFAS scores, 73 patients (55.0%) were classified as having good AOFAS-defined postoperative functional recovery (AOFAS ≥75), whereas 59 patients (45.0%) were classified as having poor AOFAS-defined postoperative functional recovery (AOFAS <75).

### Baseline characteristics between good and poor recovery groups

According to the AOFAS classification, the 132 patients were divided into the good-recovery group (AOFAS score ≥75, *n* = 73) and the poor-recovery group (AOFAS score <75, *n* = 59). Because several categorical variables contained zero or near-zero cells, between-group comparisons of categorical variables were recalculated using Fisher's exact test for 2 × 2 contingency tables and the Fisher-Freeman-Halton exact test for R × C contingency tables (Supplementary Table S3). After recalculation using exact tests, significant between-group differences remained for sprain count (*P* = 0.002), anterior ankle osteophyte formation (*P* < 0.001), syndesmotic injury (*P* < 0.001), Outerbridge cartilage damage grade (*P* < 0.001), MRI-derived ATFL remnant thickness (*P* < 0.001), and CFL/ATFL angle (*P* < 0.001). In contrast, no significant differences were observed for sex (*P* = 1.000), age group (*P* = 0.468), Beighton score (*P* = 0.167), injury duration category (*P* = 0.923), MRI-based subchondral bone marrow edema grade (*P* = 0.194), or MRI-based joint effusion grade (*P* = 0.093). These findings suggest that repeated ankle sprains, degenerative intra-articular changes, syndesmotic involvement, and abnormal ligament alignment may be associated with AOFAS-defined poor postoperative functional recovery in patients with CLAI.

### Univariate analysis of clinical characteristics according to postoperative recovery outcomes

Univariate logistic regression analysis was performed to identify variables associated with AOFAS-defined postoperative functional recovery. Among the 132 patients, several factors were significantly associated with outcome classification (Supplementary Table S4), including sprain count (*P* = 0.002), osteophytes (*P* < 0.001), syndesmotic injury (*P* = 0.049), cartilage grade (*P* < 0.001), MRI-derived lesion diameter (*P* = 0.001), CFL/ATFL angle (*P* < 0.001), and joint effusion grade (*P* = 0.035). Notably, osteophyte formation (OR = 7.873), Outerbridge grade II cartilage damage, defined as fibrillation or superficial fissures not extending to the subchondral bone (OR = 56.000), thin or absent ATFL remnant with a diameter <1.0 mm (OR = 8.455), and a CFL/ATFL angle <70° (OR = 13.636) showed the strongest associations with poor AOFAS-defined postoperative functional recovery. In contrast, no significant associations were observed for gender (*P* = 0.806), age group (*P* = 0.843), Beighton score (*P* = 0.177), injury duration (*P* = 0.726), or bone marrow edema grade (*P* = 0.926). These findings suggest that structural abnormalities of the ankle joint and lateral ligament complex, particularly osteophyte formation, cartilage injury, ATFL remnant insufficiency, and abnormal CFL/ATFL alignment, may be more closely associated with poor postoperative functional recovery than general demographic characteristics in this cohort.

### Identification of independent risk factors using LASSO and logistic regression

**Figure 2:**
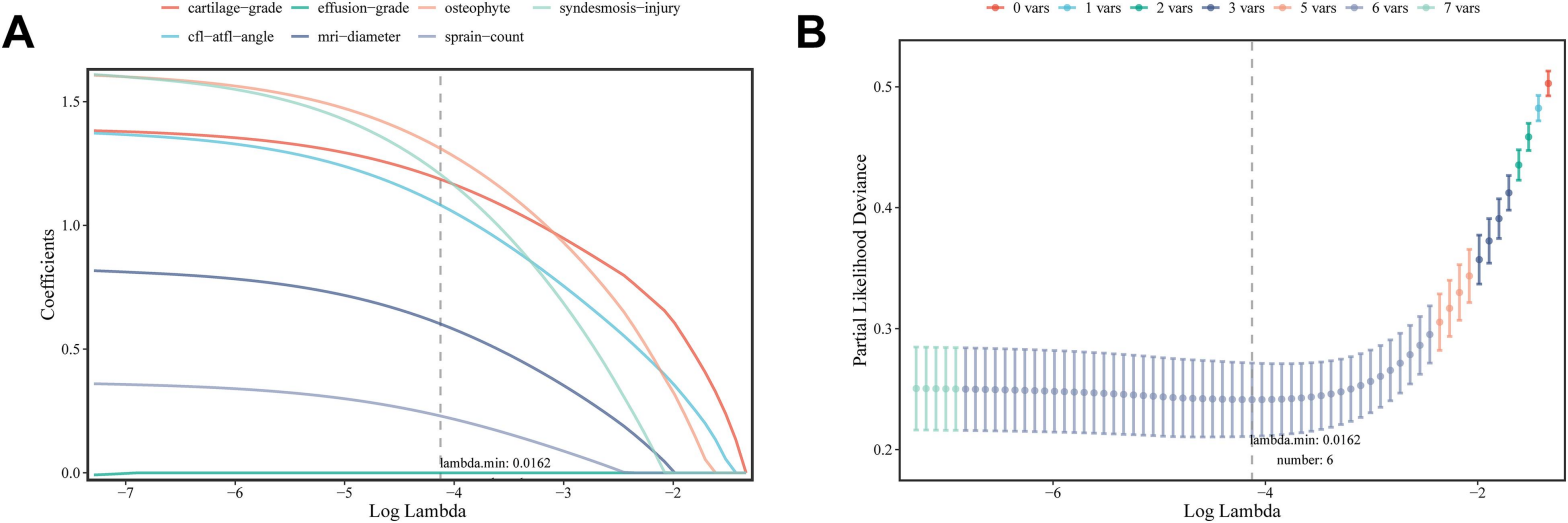
Least absolute shrinkage and selection operator (LASSO) regression for optimal feature selection in postoperative outcomes in chronic lateral ankle instability (CLAI) surgery. **(A)** Cross-validation curve for selecting the optimal penalty parameter (*λ*) using binomial deviance as the criterion. The *x*-axis represents log(*λ*), and the *y*-axis represents the partial likelihood deviance. **(B)** LASSO coefficient profiles of candidate predictors. The vertical dashed line corresponds to the optimal *λ* value (lambda.min = 0.0162) determined by 10-fold cross-validation, at which six clinical predictor domains with non-zero coefficients were retained.

To identify the most relevant predictors of postoperative recovery, LASSO regression with 10-fold cross-validation was performed. The optimal penalty parameter was determined as *λ* = 0.0162 (Figure 2A). Six variables with non-zero coefficients were retained in the model: sprain count, anterior ankle osteophyte formation, syndesmotic injury, talar cartilage damage grade, MRI-derived ATFL remnant diameter, and CFL/ATFL angle (Figure 2B). These selected variables were subsequently entered into the multivariable logistic regression model for further analysis. The final multivariable logistic regression model identified six clinical predictor domains that were independently associated with AOFAS-defined poor postoperative functional recovery. These predictors included sprain count (OR = 1.439, *P* = 0.043), osteophytes (OR = 5.951, *P* = 0.004), syndesmosis injury (OR = 5.272, *P* = 0.007), cartilage damage characterized by fibrillation or superficial cracks not extending to the subchondral bone (Outerbridge grade II; OR = 23.928, *P* = 0.017), ATFL remnant thin or absent with a diameter <1.0 mm (OR = 5.256, *P* = 0.029), and a CFL/ATFL angle <70° (OR = 11.598, *P* = 0.003) were significantly associated with AOFAS-defined poor postoperative functional recovery. Among these, cartilage damage with fibrillation or superficial cracks (Outerbridge grade II) exhibited the strongest association (OR = 23.928) (Supplementary Table S4). These findings highlight that specific structural and anatomical abnormalities, particularly cartilage damage, may contribute to AOFAS-defined poor postoperative functional recovery.

### Nomogram construction for predicting postoperative outcomes

**Figure 3:**
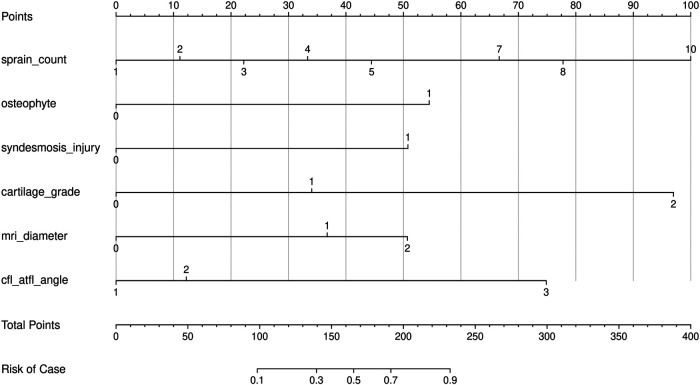
Construction of the nomogram model for predicting postoperative recovery based on the American orthopedic foot and ankle society (AOFAS) score. The nomogram integrates six clinical features, including sprain count, osteophyte, syndesmosis injury, cartilage damage grade, magnetic resonance imaging (MRI) diameter, and calcaneofibular ligament/anterior talofibular ligament angle (CFL/ATFL) angle. Each predictor was assigned a point value based on its contribution to the outcome.

Based on the multivariable logistic regression results, a predictive nomogram was constructed to estimate the probability of poor postoperative functional recovery in patients with CLAI (Figure 3). The model incorporated six clinical predictor domains: sprain count, anterior ankle osteophyte formation, syndesmotic injury, talar cartilage damage grade, MRI-derived ATFL remnant diameter, and CFL/ATFL angle. Each predictor was assigned a corresponding point value according to its regression coefficient. The total score was calculated by summing the points assigned to each variable and was then mapped to the predicted probability of poor postoperative recovery. Among the included predictors, sprain count contributed the greatest weight to the total score, followed by CFL/ATFL angle and talar cartilage damage grade. MRI-derived ATFL remnant diameter, syndesmotic injury, and osteophyte formation each contributed moderately. The total point scale ranged from 0 to 400, corresponding to an estimated risk probability of approximately 0.1 to 0.9. Higher cumulative scores were associated with a greater likelihood of poor AOFAS-defined postoperative functional recovery. This nomogram may provide a promising tool for individualized preoperative risk assessment in patients undergoing surgery for CLAI.

### Internal validation and predictive performance of the nomogram

**Figure 4:**
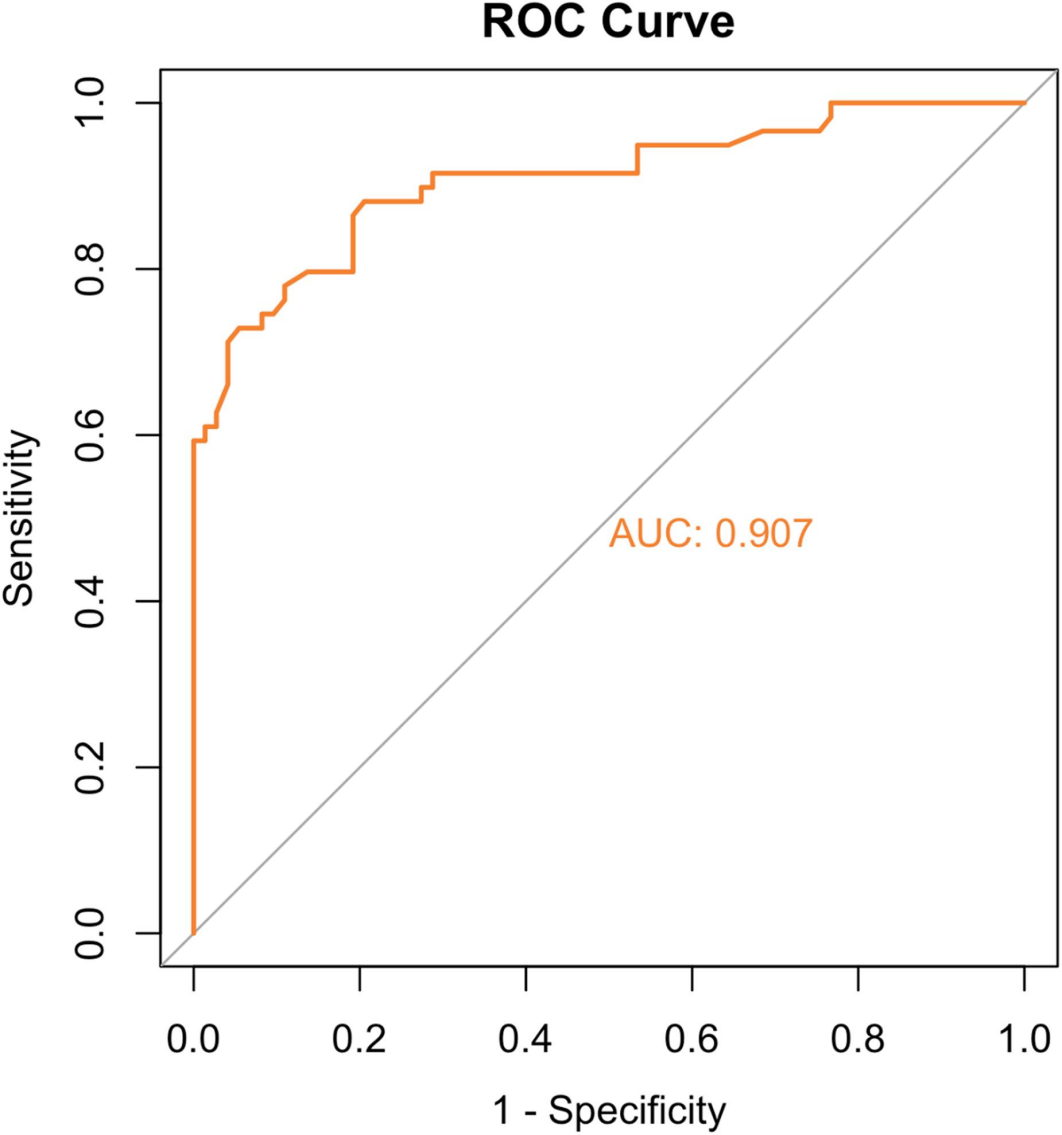
Receiver operating characteristic (ROC) curve analyses for evaluating the discriminatory power of the nomogram model.

**Figure 5:**
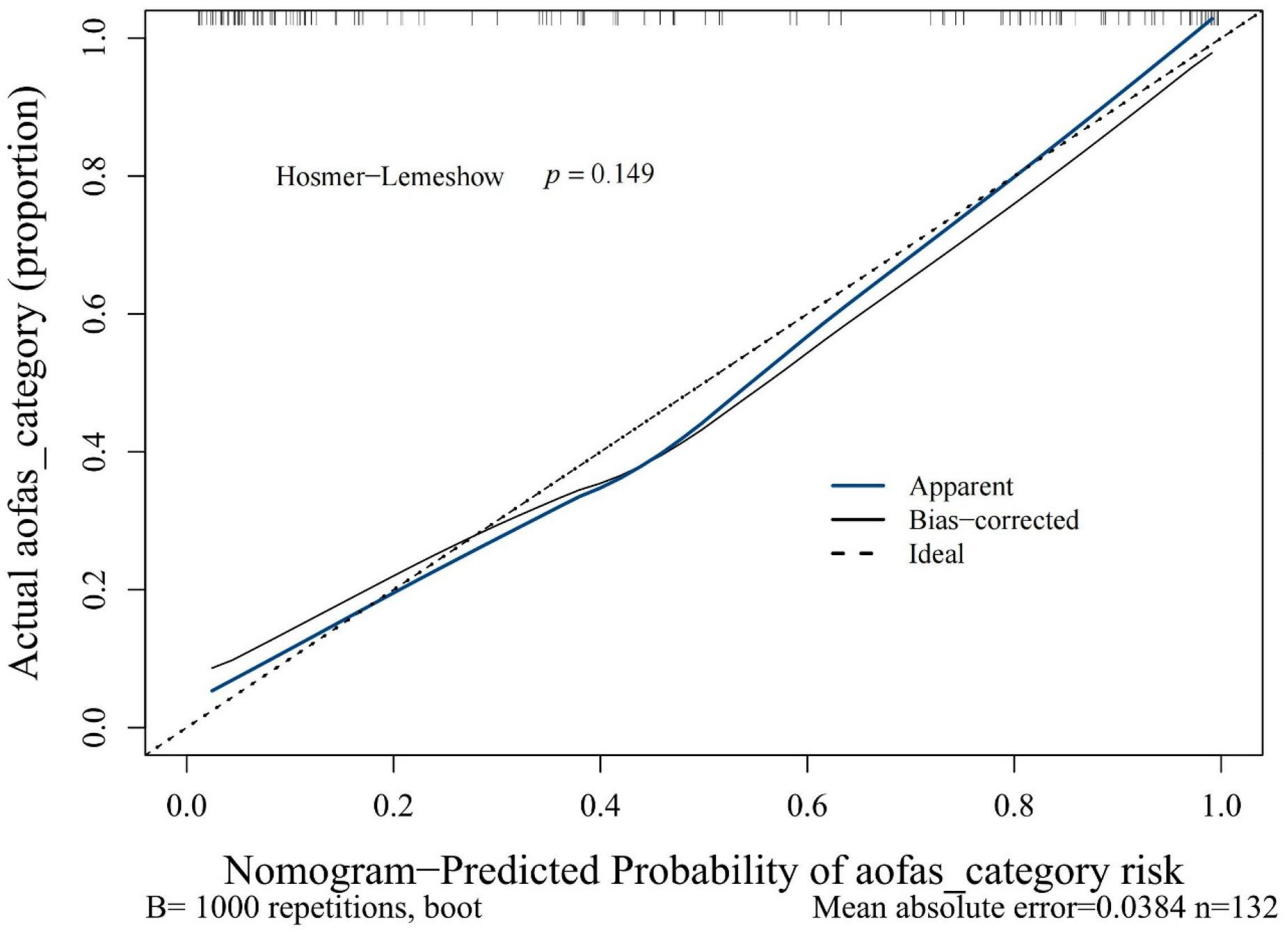
Calibration curve of the nomogram for predicting postoperative recovery. The calibration plot evaluates the agreement between the predicted and actual probabilities of postoperative recovery. The *x*-axis represents the predicted risk, and the *y*-axis shows the observed proportion. The dashed diagonal line represents perfect prediction (ideal performance), while the blue and black lines represent the apparent and bias-corrected performance, respectively, based on 1,000 bootstrap resamples.

**Figure 6:**
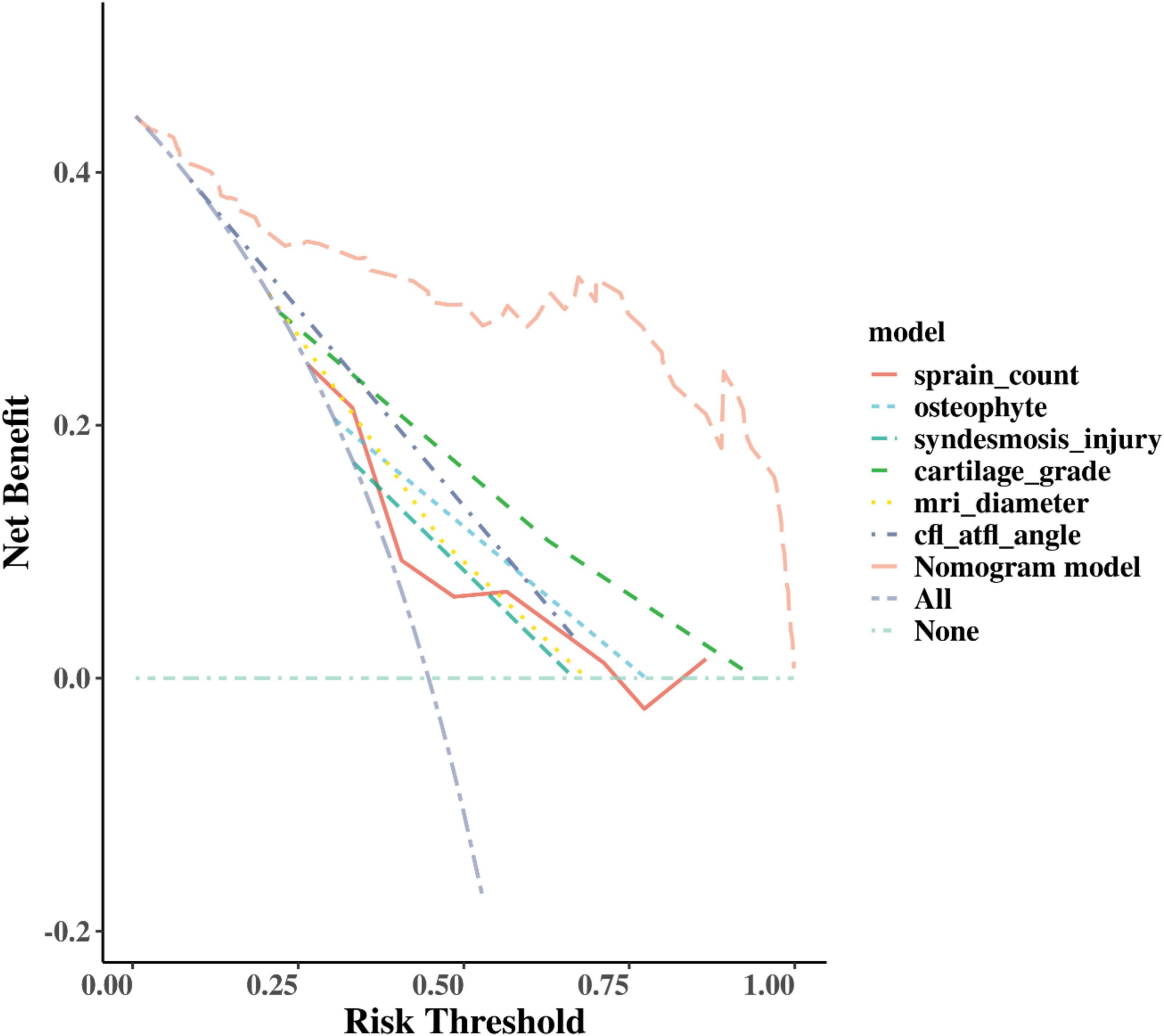
Decision curve analysis (DCA) for evaluating the clinical utility of the nomogram model. The DCA curve illustrates the net clinical benefit of using the nomogram to predict postoperative recovery across a range of threshold probabilities. The nomogram model (brown dashed line) showed a higher net benefit than any single predictor, as well as the “treat-all” or “treat-none” strategies, across most risk thresholds.

The predictive performance of the nomogram was evaluated in terms of discrimination, calibration, and clinical utility. ROC analysis showed that the nomogram had acceptable apparent discrimination in the development cohort, with an apparent AUC of 0.907 (95% CI: 0.854–0.960) (Figure 4). The optimal cutoff value based on the Youden index was 0.379, with a sensitivity of 0.881, specificity of 0.795, positive predictive value of 0.776, and negative predictive value of 0.892. Internal validation using 1,000 bootstrap resamples showed a mean AUC optimism of 0.031, resulting in an optimism-corrected AUC of 0.876 (Supplementary Table S5). These findings indicate that the model retained acceptable discriminative performance after correction for optimism, although the corrected performance was lower than the apparent estimate. Calibration analysis showed that the apparent calibration intercept and slope were 0 and 1, respectively. After bootstrap correction, the calibration intercept was −0.0752 and the calibration slope was 0.752. The Hosmer–Lemeshow goodness-of-fit test showed no significant lack of fit between the predicted and observed outcomes (*χ*^2^ = 12.040, *P* = 0.149). The mean absolute error of the calibration curve was 0.0384 (Figure 5; Supplementary Table S6), suggesting acceptable agreement between predicted and observed probabilities. However, the optimism-corrected calibration slope below 1 indicated potential overfitting, which may be related to the limited sample size and sparse data in some predictor categories. The DCA showed that the nomogram provided a higher net benefit across a range of threshold probabilities than any single predictor and the “treat-all” or “treat-none” strategies (Figure 6). The effective complexity of the final model was further assessed. Although the nomogram included six clinical predictor domains, the final logistic regression model estimated nine non-intercept coefficients after accounting for dummy variables generated from three-level categorical predictors. With 59 AOFAS-defined poor-recovery events, the effective events-per-variable was 6.56 (Supplementary Table S7). This value was below the conventional threshold of 10 events per variable, indicating a limited effective sample size relative to model complexity. This finding was consistent with the internal validation results, particularly the bootstrap-corrected calibration slope of 0.752, which suggested potential model overfitting. Overall, the nomogram demonstrated acceptable discrimination, calibration, and potential clinical utility in the development cohort. Nevertheless, further external validation is required before routine clinical application.

### Sensitivity analysis using Firth penalized likelihood logistic regression

Because several predictors contained sparse categories, particularly Outerbridge grade II cartilage damage, Firth penalized likelihood logistic regression was performed as a sensitivity analysis. Overall, the Firth-corrected estimates showed the same direction of association as the conventional multivariable logistic regression model, although the ORs were generally attenuated. For example, the association between Outerbridge grade II cartilage damage and AOFAS-defined poor postoperative functional recovery decreased from an OR of 23.928 in the conventional logistic model to a Firth-corrected OR of 12.700, while remaining statistically significant. Similarly, anterior ankle osteophyte formation, syndesmotic injury, thin or absent ATFL remnant, and CFL/ATFL angle <70° remained associated with poor postoperative functional recovery after Firth correction. Sprain count showed an attenuated and borderline association after Firth correction. These findings suggest that the main associations were directionally stable, but the absolute magnitude of the conventional OR estimates, particularly for sparse categories, should be interpreted cautiously. The Firth-corrected estimates were as follows: sprain count, OR = 1.369, 95% CI: 0.994–1.884, *P* = 0.054; anterior ankle osteophyte formation, OR = 4.792, 95% CI: 1.528–15.034, *P* = 0.007; syndesmotic injury, OR = 4.237, 95% CI: 1.378–13.028, *P* = 0.012; Outerbridge grade I cartilage damage vs. grade 0, OR = 2.689, 95% CI: 0.880–8.215, *P* = 0.083; Outerbridge grade II cartilage damage vs. grade 0, OR = 12.700, 95% CI: 1.455–110.828, *P* = 0.021; MRI-derived ATFL remnant thickness grade 1 vs. grade 0, OR = 2.800, 95% CI: 0.757–10.353, *P* = 0.123; thin or absent ATFL remnant <1.0 mm vs. normal thickness, OR = 4.302, 95% CI: 1.081–17.122, *P* = 0.038; CFL/ATFL angle 70°–90° vs. ≥91°, OR = 1.321, 95% CI: 0.247–7.060, *P* = 0.745; and CFL/ATFL angle <70° vs. ≥91°, OR = 7.918, 95% CI: 1.867–33.575, *P* = 0.005 (Supplementary Table S8).

## Discussion

Postoperative functional assessment is essential for evaluating surgical outcomes in patients with CLAI. The AOFAS Ankle-Hindfoot score is widely used in foot and ankle surgery and has been applied in studies assessing Broström-type repair, suture-tape augmentation, and other ligament stabilization procedures for CLAI. By integrating pain, functional capacity, and alignment, this score provides a clinically interpretable measure of ankle-hindfoot recovery, with higher scores indicating better postoperative function (21, 23, 24). In the present study, 132 patients with CLAI undergoing arthroscopic Broström repair with suture-tape augmentation were retrospectively analyzed. Based on the prespecified AOFAS Ankle-Hindfoot score cutoff, 73 patients achieved good AOFAS-defined recovery, whereas 59 patients had poor AOFAS-defined recovery. Among the prespecified candidate variables, LASSO logistic regression and multivariable logistic regression identified six variables associated with poor recovery: number of ankle sprains, preoperative ATFL remnant thickness, distal tibiofibular syndesmotic injury, anterior ankle osteophyte formation, talar cartilage injury, and an ATFL-CFL angle <70°. The nomogram showed high apparent discrimination and acceptable calibration in the development cohort, and DCA suggested potential clinical utility. Our study should also be interpreted in the context of the recently published model by Chen et al., who developed a nomogram for functional outcomes after all-inside arthroscopic ATFL repair in chronic ankle instability and identified increasing age, female sex, non-sports-related ankle sprain, and lower-leg cast immobilization as predictors of poorer outcomes (25). Methodologically, both studies used a regression-based prediction framework and aimed to translate multiple clinical variables into an individualized prognostic tool. This similarity supports the growing clinical relevance of predictive modeling in the management of chronic ankle instability. However, several important differences distinguish the present study from the previous model. First, the outcome assessment differed, as the previous study used the Karlsson Ankle Functional Score, whereas our study classified postoperative recovery according to the AOFAS Ankle-Hindfoot score. Then, and most importantly, the predictors identified in the two models were distinct. The previous model emphasized demographic and perioperative factors, including age, sex, injury mechanism, and immobilization method, whereas our model identified structural and anatomical predictors, including sprain count, osteophyte formation, syndesmosis injury, Outerbridge grade II cartilage damage, thin or absent ATFL remnant, and CFL/ATFL angle <70°. These differences suggest that postoperative recovery after CLAI surgery may be influenced by procedure-specific and anatomy-specific factors. Therefore, the added value of the present model lies in its integration of MRI-derived ligament morphology, ligament alignment, syndesmotic status, and arthroscopic cartilage findings, which may provide more direct information regarding local joint degeneration and repairability. Meanwhile, the outcome definition of this study should be interpreted with caution. Although the AOFAS Ankle-Hindfoot score is widely used in foot and ankle surgery and offers a clinically interpretable assessment of pain, function, and alignment, it is not a validated CAI-specific patient-reported outcome measure. Instruments such as the Cumberland Ankle Instability Tool (CAIT), Identification of Functional Ankle Instability (IdFAI) score, Foot and Ankle Ability Measure (FAAM), and Patient-Reported Outcomes Measurement Information System (PROMIS) measures may better capture subjective instability, giving-way episodes, sport-related limitations, psychological burden, and patient-perceived recovery, but these measures were not systematically collected because of the retrospective design. Therefore, we could not determine whether AOFAS-defined poor recovery reflected persistent or recurrent CAI. An AOFAS score <75 may indicate residual instability, but may also result from pain, osteochondral injury, anterior ankle impingement, restricted range of motion, activity limitation, or other postoperative factors. Moreover, the 75-point cutoff was not derived from an anchor-based minimal clinically important difference (MCID) or patient acceptable symptom state (PASS) threshold. Accordingly, the present model should be interpreted as predicting a broader composite endpoint of AOFAS-defined poor ankle-hindfoot functional recovery, rather than residual instability, persistent CAI, failure of ligament stabilization, or comprehensive CAI-specific treatment failure. Future prospective studies should incorporate validated CAI-specific PROMs, objective instability tests, pain assessment, imaging findings, and clinically anchored MCID or PASS thresholds to improve model validity and clinical interpretability.

Sprain frequency is an important indicator for assessing both the severity of ankle injury and the likelihood of developing chronic instability. Numerous studies have shown that recurrent sprains are not only a hallmark clinical symptom of CAI, but also an important factor influencing surgical outcomes (26, 27). In a cohort of 30 South African professional ballet dancers, the prevalence of CAI was reported to be 73.3%, with 83.3% of affected dancers having experienced at least one severe ankle sprain (28). A retrospective study involving 38 patients with CAI who underwent modified Broström-Gould (MBG) surgery has reported that patients with three or more preoperative sprains had significantly less improvement in AOFAS scores than those with fewer than three sprains (*P* < 0.05). Moreover, their motor functional recovery is notably delayed at 1 year after surgery (29). Understanding the relationship between sprain frequency and postoperative functional recovery is essential for optimizing surgical strategies and rehabilitation protocols. Our findings showed that an increased number of sprains independently predicted a poorer prognosis. In addition to recurrent sprains, concomitant structural injuries may further compromise postoperative recovery. Distal tibiofibular syndesmotic injuries account for approximately 1%–18% of ankle sprains and occur more frequently in contact sports (30). A study evaluating 118 patients with CLAI who underwent Broström-Gould surgery reveals that severe tibiofibular syndesmosis widening (≥4 mm) is associated with delayed return to work and recreational sports, a lower rate of return to preinjury sports, and a higher recurrence rate of sprain (31). In line with these findings, the present study showed that concomitant distal tibiofibular syndesmotic injury was associated with an increased risk of poor postoperative function. Anatomical studies have demonstrated that the ATFL and CFL form an angle in the coronal plane, typically ranging from 100° to 120°. This angle determines the direction of the resultant force and the relative contribution of each ligament to ankle stability (32, 33). An abnormal ATFL-CFL angle often indicates a severe tear of the anterior talofibular ligament, which may compromise surgical outcomes by disrupting the tension balance after ligament repair or reconstruction (34, 35). Postoperative imaging evaluations have shown that patients whose ATFL-CFL angle returns to 105°–115° after arthroscopic reconstruction exhibit better postoperative balance function and muscle strength recovery (36). The present study showed that an abnormal ATFL–CFL angle was associated with poor postoperative functional recovery, suggesting that preoperative alteration of ligament orientation may serve as an important imaging indicator for identifying patients at higher risk of unsatisfactory outcomes. A thinner ATFL stump on preoperative MRI is closely associated with poorer functional outcomes after arthroscopic repair, as well as an increased risk of recurrent instability and re-tear (37, 38). In addition, quantitative MRI signal analysis can characterize histological degeneration and mechanical deterioration of the ATFL stump. An increased signal intensity ratio of the ATFL may indicate fibrosis or myxoid degeneration and is closely related to intraoperative repairability and postoperative return to exercise capacity (39). Furthermore, T2 mapping provides an objective assessment of ATFL tissue quality. In patients with CAI, ATFL T2 values are significantly increased and correlate with mechanical instability, suggesting their potential value as quantitative indicators for perioperative risk stratification and outcome prediction (40). Kamada et al. also have reported that a normal ATFL appears continuous and uniform in thickness, whereas morphological changes such as thinning, striation, and signal abnormalities are common in CAI and associated with poor surgical outcomes (41).

Another critical factor influencing postoperative prognosis is the extent of cartilage injury. Cartilage damage is not only an important contributor to pain and dysfunction in CAI, but also affects surgical outcomes and increases the risk of PTOA (42). A systematic review and meta-analysis reports that approximately one-third of patients with CLAI have concomitant cartilage or osteochondral lesions (43). Wei et al. have conducted a prospective randomized study involving patients with CLAI and osteochondral lesions of the talus. They found that single-stage arthroscopic microfracture combined with modified Broström-Gould repair is not inferior to staged surgery at mid-term follow-up and provided better short-term functional recovery, suggesting that simultaneous restoration of ligament stability and cartilage lesion management may be feasible in selected patients (44). However, the management of minor cartilage lesions remains controversial. A randomized controlled trial illustrates that, among patients with CAI and small talar chondral defects, additional microfracture did not improve the AOFAS score, Self-reported Foot and Ankle Score (SEFAS), Karlsson Ankle Functional Score, pain outcomes, or complication rates at 2 years compared with isolated ligament reconstruction, but delayed early functional recovery (45). These findings suggest that minor cartilage lesions should not be treated uniformly, and rehabilitation timing should be individualized according to lesion size, symptoms, and restoration of ankle stability. In the present study, although cartilage injuries were limited to Outerbridge grade II on arthroscopic assessment, they were still identified as an independent risk factor for impaired postoperative ankle functional recovery. Anterior ankle osteophyte formation represents an adaptive response to joint degeneration and is closely associated with abnormal biomechanics resulting from CAI. The presence of anterior ankle osteophytes can markedly affect postoperative functional recovery in patients with CAI (46, 47). Studies have shown that CAI surgery combined with osteophyte resection can improve ankle dorsiflexion by 10°–15° and reduce Visual Analog Scale (VAS) pain scores by 4–5 points at 6 months after surgery (48). In terms of long-term prognosis, patients with anterior ankle osteophytes still have a higher risk of postoperative PTOA than those without osteophytes. The presence of osteophytes itself indicates joint degeneration, and even after complete resection, underlying cartilage damage and biomechanical abnormalities may persist, potentially leading to further joint deterioration (42, 49). Overall, our findings suggest that poor postoperative recovery in patients with CLAI may be associated with multiple preoperative and intra-articular factors, including recurrent sprains, syndesmotic injury, abnormal ATFL–CFL angle, reduced ATFL remnant thickness, cartilage injury, and anterior ankle osteophytes. By incorporating these factors, we developed an internally validated nomogram to estimate the individualized risk of AOFAS-defined poor postoperative functional recovery. This model may serve as a preliminary risk-stratification tool to support preoperative assessment, individualized rehabilitation planning, and postoperative follow-up management.

The present cohort was predominantly male (86%) and mainly composed of patients aged 18–45 years (92%). Although this distribution is consistent with the epidemiological profile of sports-related CLAI, it may limit the generalizability of the nomogram to females, adolescents, and older adults. Previous studies have similarly shown that ankle instability and sports-related ligament injuries are frequently observed in young and physically active populations. For example, a systematic literature search of PubMed and Web of Science identified 3,804 participants aged 15–32 years, including soldiers, students, athletes, and other physically active individuals with a history of ankle sprain (50). Likewise, a cross-sectional study of 712 patients with anterior cruciate ligament rupture reported that most patients were male (93.1%) and aged 15–30 years (80.2%) (51). These findings support the relevance of studying young, physically active patients, but they also highlight that such cohorts may not fully represent the broader clinical spectrum of CLAI. Sex- and age-related differences may influence both disease presentation and postoperative recovery in patients with CLAI. Female athletes participating in high-risk sports for ankle sprains are particularly susceptible to chronic ankle instability (52), and may differ from male patients in ligamentous laxity, hormonal influences on tissue healing, neuromuscular control, and biomechanical alignment. These factors may alter the predictive weight of anatomical and imaging variables such as the CFL/ATFL angle and ATFL remnant thickness identified in the present study. Age may also modify postoperative prognosis. In patients with CLAI, increasing age has been inversely correlated with postoperative clinical outcomes, with older patients showing significantly lower preoperative and postoperative functional and pain-related scores than younger patients (53). Moreover, adolescents with open physes and older adults with age-related cartilage degeneration may represent distinct clinical subgroups in whom predictors such as Outerbridge grade and osteophyte formation carry different prognostic implications. Therefore, external validation in larger and more demographically diverse cohorts is warranted before this nomogram can be broadly applied to females, adolescents, and older adults. In addition, the wide confidence interval observed for Outerbridge grade II cartilage damage (OR = 23.928, 95% CI: 2.61–643.18) warrants careful interpretation. This instability most likely reflects the limited number of events within this specific subgroup, given the overall sample size of 132 patients and 59 poor-recovery outcomes. When a categorical predictor is associated with a small number of observations concentrated heavily in one outcome category, maximum likelihood estimation can produce inflated point estimates and correspondingly wide confidence intervals — a phenomenon related to quasi-complete separation. While LASSO regression was used for variable selection to mitigate overfitting prior to multivariable modeling, LASSO penalization primarily addresses coefficient shrinkage during the selection phase; it does not eliminate the small-sample variance inherent in the final unpenalized logistic regression estimates for retained variables. Consequently, while cartilage damage retained statistical and clinical significance across bootstrap resampling, the precision of this specific point estimate should be interpreted with caution, and the magnitude of the OR should not be over-interpreted as a precise effect size.

Several limitations should be acknowledged. First, this was a single-center retrospective model-development study with a relatively limited sample size, which may have introduced selection bias and reduced the generalizability of the findings. Although 59 patients experienced AOFAS-defined poor postoperative functional recovery, the number of outcome events may still have been insufficient to develop a stable prediction model incorporating six clinical predictor domains. This limitation is further reflected by the wide confidence intervals observed for several predictors, suggesting imprecision and possible instability of the regression estimates, particularly in the presence of sparse data in some predictor categories. Second, although internal validation was performed using 1,000 bootstrap resamples, the optimism-corrected AUC was lower than the apparent AUC, and the bootstrap-corrected calibration slope was below 1, indicating potential model overfitting. Therefore, the present nomogram should be interpreted as a development-stage prediction model rather than a definitive clinical decision-making tool. Future multicenter studies with larger and more diverse cohorts are needed to externally validate the model, evaluate its generalizability across different clinical settings, and update or recalibrate the model when necessary. Third, postoperative rehabilitation adherence was not quantitatively recorded. Although all patients were instructed to follow a standardized rehabilitation protocol and underwent routine postoperative follow-up, individual differences in actual adherence, exercise intensity, weight-bearing progression, and return-to-sport timing may have influenced functional recovery. Rehabilitation adherence represents a potential residual confounder that could not be adjusted for in the present retrospective model. In our future prospective studies, we will incorporate objective adherence measures, such as rehabilitation logs, therapist-supervised attendance records, mobile-based monitoring, or wearable activity data, to better evaluate the effect of rehabilitation compliance on postoperative outcomes. Fourth, all operations were performed by a single experienced foot and ankle surgeon. This design reduced inter-surgeon variability and improved the consistency of surgical technique and perioperative management. However, it also limited our ability to assess the potential effects of surgeon volume, surgical experience, and learning curve on postoperative outcomes. Accordingly, the generalizability of the present nomogram to other surgeons, lower-volume centers, or institutions using different operative techniques should be interpreted with caution.

## Conclusion

This study identified six variables associated with AOFAS-defined poor postoperative functional recovery in patients with CLAI undergoing arthroscopic Broström repair with suture-tape augmentation. Based on these variables, a development-stage nomogram was constructed and internally validated for individualized risk estimation. The model demonstrated acceptable discrimination, calibration, and potential clinical utility in the development cohort. However, further external validation and possible recalibration in larger, multicenter cohorts are required before routine clinical application.

## Data Availability

The original contributions presented in the study are included in the article/Supplementary Material, further inquiries can be directed to the corresponding author.
