# Supplementary material for: A LASSO regression-driven nomogram to predict functional outcomes after surgery for chronic lateral ankle instability
Source: Front Surg. 2026 Aug 5;13:1758259. doi: 10.3389/fsurg.2026.1758259 (PMC13485904; doi:10.3389/fsurg.2026.1758259)
Supplement: Supplementary file 1 [file Table1.docx]

Table S1 Variable classification and coding used for statistical analysis

| **Variable** | **Value** | **Assignment statement** |
| --- | --- | --- |
| **age (years)** | 25.35±7.42（16~65） |  |
| **gender**  male  female | 113  19 | female=0 ，male=1 |
| **Anterior ankle osteophyte**  no  yes | 92  40 | no=0 ，yes=1 |
| **Associated distal tibiofibular injury**  no  yes | 89  43 | no=0 ，yes=1 |
| **Cartilage classification**  normal  Ⅰ  Ⅱ  Ⅲ  Ⅳ | 75  42  15  0  0 | =0  =1  =2  =3  =4 |
| **MRI diameter**  normal: diameter = 1.0–3.2 mm  thickened: diameter > 3.2 mm  thinned or absent: diameter < 1.0 mm | 41  52  39 | =0  =1  =2 |
| **Grading of subchondral bone marrow edema**  no subchondral edema  mild subchondral bone edema  severe subchondral bone edema | 53  44  35 | =0  =1  =2 |
| **Preoperative MRI-based grading of joint effusion**  Grade 0: minimal physiological fluid (normal)  Grade 1: <50% of maximal capsular distention  Grade 2: ≥50% of maximal capsular distention | 47  57  28 | =0  =1  =2 |
| **CFL/ATFL angle**  ≥91°  70°-90°  ＜70° | 29  33  70 | =1  =2  =3 |
| AOFAS score | good outcome ≥75  poor outcome <75 | =0  =1 |

Continuous variables (e.g., age) were expressed as mean ± standard deviation. Categorical variables were binary encoded (0/1). Ordinal variables were assigned consecutive integers based on severity thresholds. The AOFAS scores were dichotomized into "good" (≥75) and "poor" (<75).

Table S2. Baseline characteristics of the overall cohort (N = 132)

**Variables	N = 132**

**gender**

0	19 (14%)

1	113 (86%)

**age group**

| 1 (＜18 years) | 2 (1.5%) |
| --- | --- |
| 2 (18–45 years) | 121 (92%) |
| 3 (46–60 years) | 7 (5.3%) |
| 4 (>60 years) | 2 (1.5%) |

**Beighton score**

| 0 | 25 (19%) |
| --- | --- |
| 1 | 27 (20%) |
| 2 | 54 (41%) |
| 3 | 19 (14%) |
| 4 | 3 (2.3%) |
| 5 | 4 (3.0%) |

**sprain count**

1	11 (8.3%)

2	38 (29%)

3	24 (18%)

4	27 (20%)

5	20 (15%)

7	2 (1.5%)

8	8 (6.1%)

10	2 (1.5%)

**injury duration**	17.2 ± 20.1

**osteophyte**

0	92 (70%)

1	40 (30%)

**syndesmosis injury**

0	89 (67%)

1	43 (33%)

**cartilage grade**

| 0 | 75 (57%) |
| --- | --- |
| 1 | 42 (32%) |
| 2 | 15 (11%) |

**MRI diameter**

| 0 | 41 (31%) |
| --- | --- |
| 1 | 52 (39%) |
| 2 | 39 (30%) |

**edema grade**

| 0 | 53 (40%) |
| --- | --- |
| 1 | 44 (33%) |
| 2 | 35 (27%) |

**effusion grade**

| 0 | 47 (36%) |
| --- | --- |
| 1 | 57 (43%) |
| 2 | 28 (21%) |

**CFL/ATFL angle**

| 1 | 29 (22%) |
| --- | --- |
| 2 | 33 (25%) |
| 3 | 70 (53%) |

**AOFAS category**

0	73 (55%)

1	59 (45%)

Values are presented as n (%) for categorical variables and mean ± standard deviation (SD) for continuous variables.

Table S3. Exact tests used for between-group comparisons of categorical variables

| **Variable** | **Contingency table dimension** | **Exact P value** | **Test method** |
| --- | --- | --- | --- |
| Sex | 2 × 2 | 1.000 | Fisher’s exact test |
| Age group | 4 × 2 | 0.468 | Fisher-Freeman-Halton exact test |
| Beighton score | 6 × 2 | 0.167 | Fisher-Freeman-Halton exact test |
| Sprain count | 8 × 2 | 0.002 | Fisher-Freeman-Halton exact test |
| Injury duration | 15 × 2 | 0.923 | Fisher-Freeman-Halton exact test |
| Anterior ankle osteophytes | 2 × 2 | <0.001 | Fisher’s exact test |
| Syndesmotic injury | 2 × 2 | <0.001 | Fisher’s exact test |
| Outerbridge cartilage damage grade | 3 × 2 | <0.001 | Fisher-Freeman-Halton exact test |
| MRI-derived ATFL remnant thickness | 3 × 2 | <0.001 | Fisher-Freeman-Halton exact test |
| MRI-based subchondral bone marrow edema grade | 3 × 2 | 0.194 | Fisher-Freeman-Halton exact test |
| MRI-based joint effusion grade | 3 × 2 | 0.093 | Fisher-Freeman-Halton exact test |
| CFL/ATFL angle | 3 × 2 | <0.001 | Fisher-Freeman-Halton exact test |

Firth penalized likelihood logistic regression was performed as a sensitivity analysis. The corresponding 95% confidence intervals (CIs) and P values were derived using Wald-type approximations. Events per variable/parameter (EPV) was calculated based on the nine non-intercept coefficients actually estimated in the final model, rather than solely on the six clinical predictor domains. For between-group comparisons of categorical variables, Fisher’s exact test was used for 2 × 2 contingency tables, whereas the Fisher–Freeman–Halton exact test was used for r × c contingency tables.

Table S4. Univariate and multivariable logistic regression analyses of factors associated with AOFAS-defined poor postoperative functional recovery

| **Variable** | **Univariate OR (95% CI)** | **Univariate P-value** | **Multivariable OR (95% CI)** | **Multivariable P-value** |
| --- | --- | --- | --- | --- |
| **Sex** |  |  |  |  |
| 0 |  |  | — | — |
| 1 | 1.131 (0.426–3.122) | 0.806 | — | — |
| **Age group** |  |  |  |  |
| 1 |  |  | — | — |
| 2 | 0.754 (0.029–19.355) | 0.843 | — | — |
| 3 | 1.333 (0.041–44.449) | 0.858 | — | — |
| 4 | Not estimable | — | — | — |
| Beighton score | 0.813 (0.596–1.093) | 0.177 | — | — |
| Sprain count | 1.378 (1.132–1.718) | 0.002 | 1.439 (1.024–2.084) | 0.043 |
| Injury duration | 1.003 (0.985–1.021) | 0.726 | — | — |
| **Osteophyte** |  |  |  |  |
| 0 |  |  |  |  |
| 1 | 7.873 (3.431–19.619) | <0.001 | 5.951 (1.829–21.613) | 0.004 |
| **Syndesmotic injury** |  |  |  |  |
| 0 |  |  |  |  |
| 1 | 4.000 (1.892–8.767) | <0.001 | 5.272 (1.639–18.981) | 0.007 |
| **Cartilage damage grade** |  |  |  |  |
| 0 |  |  |  |  |
| 1 | 5.906 (2.620–13.909) | <0.001 | 3.048 (0.938–10.322) | 0.065 |
| 2 | 56.000 (10.253–1049.777) | <0.001 | 23.928 (2.610–643.180) | 0.017 |
| **MRI-derived ATFL remnant thickness** |  |  |  |  |
| 0 |  |  |  |  |
| 1 | 3.375 (1.372–9.018) | 0.011 | 3.328 (0.845–15.135) | 0.097 |
| 2 | 8.455 (3.063–25.641) | <0.001 | 5.256 (1.242–25.565) | 0.029 |
| **Bone marrow edema grade** |  |  |  |  |
| 0 |  |  | — | — |
| 1 | 1.038 (0.466–2.317) | 0.926 | — | — |
| 2 | 0.476 (0.190–1.147) | 0.104 | — | — |
| **Joint effusion grade** |  |  |  |  |
| 0 |  |  | — | — |
| 1 | 2.370 (1.073–5.391) | 0.035 | — | — |
| 2 | 2.133 (0.818–5.664) | 0.123 | — | — |
| **CFL/ATFL angle** |  |  |  |  |
| 1 |  |  |  |  |
| 2 | 1.683 (0.451–7.088) | 0.448 | 1.492 (0.235–10.539) | 0.672 |
| 3 | 13.636 (4.637–50.636) | <0.001 | 11.598 (2.624–70.723) | 0.003 |

Variables not included in the final multivariable model are indicated by “—”.

Table S5. Discrimination performance and internal validation of the prediction model

| **Metric** | **Value** |
| --- | --- |
| Sample size | 132 |
| Number of patients with poor outcome | 59 |
| Number of patients without poor outcome | 73 |
| Number of bootstrap resamples | 1,000 |
| Apparent AUC | 0.907 |
| 95% CI for AUC | 0.8539–0.9601 |
| Mean optimism in AUC | 0.031 |
| Optimism-corrected AUC | 0.876 |
| Optimal Youden cutoff | 0.3792 |
| Sensitivity | 0.8814 |
| Specificity | 0.7945 |
| Positive predictive value | 0.7761 |
| Negative predictive value | 0.8923 |

Table S6. Calibration performance and bootstrap validation of the prediction model

| **Calibration parameter** | **Value** |
| --- | --- |
| Number of bootstrap resamples | 1,000 |
| Hosmer–Lemeshow χ² statistic | 12.0397 |
| Hosmer–Lemeshow test P-value | 0.1494 |
| Apparent calibration intercept | 0 |
| Apparent calibration slope | 1 |
| Bootstrap-corrected calibration intercept | -0.0752 |
| Bootstrap-corrected calibration slope | 0.7522 |
| Mean absolute error of the calibration curve | 0.0384 |

Table S7. Effective model complexity and events-per-variable calculation

| **Item** | **Result** |
| --- | --- |
| Total sample size | 132 |
| Good postoperative recovery | 73 |
| AOFAS-defined poor postoperative recovery events | 59 |
| Clinical predictor domains included in the final model | 6 |
| Non-intercept coefficients/effective degrees of freedom estimated in the final model | 9 |
| Events-per-variable based on effective degrees of freedom | 6.56 |

Table S8. Sensitivity analysis comparing conventional logistic regression and Firth penalized likelihood logistic regression

| **Predictor** | **Conventional logistic OR (95% CI)** | **Conventional P value** | **Firth-corrected OR (95% CI)** | **Firth-corrected P value** |
| --- | --- | --- | --- | --- |
| Sprain count | 1.439 (1.024–2.084) | 0.043 | 1.369 (0.994–1.884) | 0.054 |
| Anterior ankle osteophytes, yes vs. no | 5.951 (1.829–21.613) | 0.004 | 4.792 (1.528–15.034) | 0.007 |
| Syndesmotic injury, yes vs. no | 5.272 (1.639–18.981) | 0.007 | 4.237 (1.378–13.028) | 0.012 |
| Outerbridge grade I cartilage damage vs. grade 0 | 3.048 (0.938–10.322) | 0.065 | 2.689 (0.880–8.215) | 0.083 |
| Outerbridge grade II cartilage damage vs. grade 0 | 23.928 (2.610–643.180) | 0.017 | 12.700 (1.455–110.828) | 0.021 |
| MRI-derived ATFL remnant thickness grade 1 vs. grade 0 | 3.328 (0.845–15.135) | 0.097 | 2.800 (0.757–10.353) | 0.123 |
| Thin or absent ATFL remnant <1.0 mm, grade 2 vs. grade 0 | 5.256 (1.242–25.565) | 0.029 | 4.302 (1.081–17.122) | 0.038 |
| CFL/ATFL angle 70°–90° vs. ≥91° | 1.492 (0.235–10.539) | 0.672 | 1.321 (0.247–7.060) | 0.745 |
| CFL/ATFL angle <70° vs. ≥91° | 11.598 (2.624–70.723) | 0.003 | 7.918 (1.867–33.575) | 0.005 |
